# A Rare Case of a Cardiac Papillary Fibroelastoma Arising in the Right Atrial Septum and Resected via Minimally Invasive Cardiac Surgery

**DOI:** 10.7759/cureus.76486

**Published:** 2024-12-27

**Authors:** Kentaro Akabane, Yoshinori Kuroda, Tetsuro Uchida

**Affiliations:** 1 Second Department of Surgery, Faculty of Medicine, Yamagata University, Yamagata, JPN

**Keywords:** cardiac papillary fibroelastoma, cardiac tumor, minimally invasive cardiac surgery, multimodality imaging, right atrial septum

## Abstract

Papillary fibroelastomas (PFEs) are rare, benign, primary cardiac tumors, typically found on the valve surfaces and more commonly on the left side of the heart, with occurrences in the right atrium even rarer. In this case, a highly mobile tumor was incidentally detected in the right atrium of an 83-year-old woman with advanced right lung cancer during preoperative transthoracic echocardiography and magnetic resonance imaging. Although the patient was asymptomatic and of advanced age, the tumor’s high mobility warranted resection. Given her low surgical tolerance, simultaneous cardiac and lung surgery was deemed too risky. Therefore, we decided to prioritize right upper lobectomy for lung cancer, followed by minimally invasive cardiac surgery (MICS) for tumor resection. The postoperative course was uneventful, and pathological examination confirmed the diagnosis of a PFE. Although PFEs are benign and often asymptomatic, especially when located on the right side of the heart, they can lead to fatal complications such as acute myocardial infarction or pulmonary embolism. Therefore, early surgical resection should be considered, particularly for highly mobile tumors. In addition, resection via MICS may be an effective option, depending on the location and morphology of the tumor.

## Introduction

Papillary fibroelastomas (PFEs) are rare, benign primary cardiac tumors [1], typically found on the valve surfaces and more commonly on the left side of the heart, with occurrences in the right atrium even rarer, at a rate of only 2% [2]. The advancement of multimodality cardiac imaging has improved the diagnostic accuracy for cardiac tumors [3]. Transthoracic echocardiography (TTE) is the primary diagnostic imaging technique for assessing tumor location and mobility, while cardiac magnetic resonance (CMR) imaging offers superior soft tissue characterization and aids in differential diagnosis. Although PFEs are often asymptomatic and detected incidentally, early surgical resection is generally recommended due to the risk of fatal embolism from tumor collapse or the release of fibrin thrombi from its surface [4]. This report describes a rare case of PFE arising from the right atrial septum, which was resected via minimally invasive cardiac surgery (MICS), focusing on diagnostic features and therapeutic interventions.

## Case presentation

**Figure 1:**
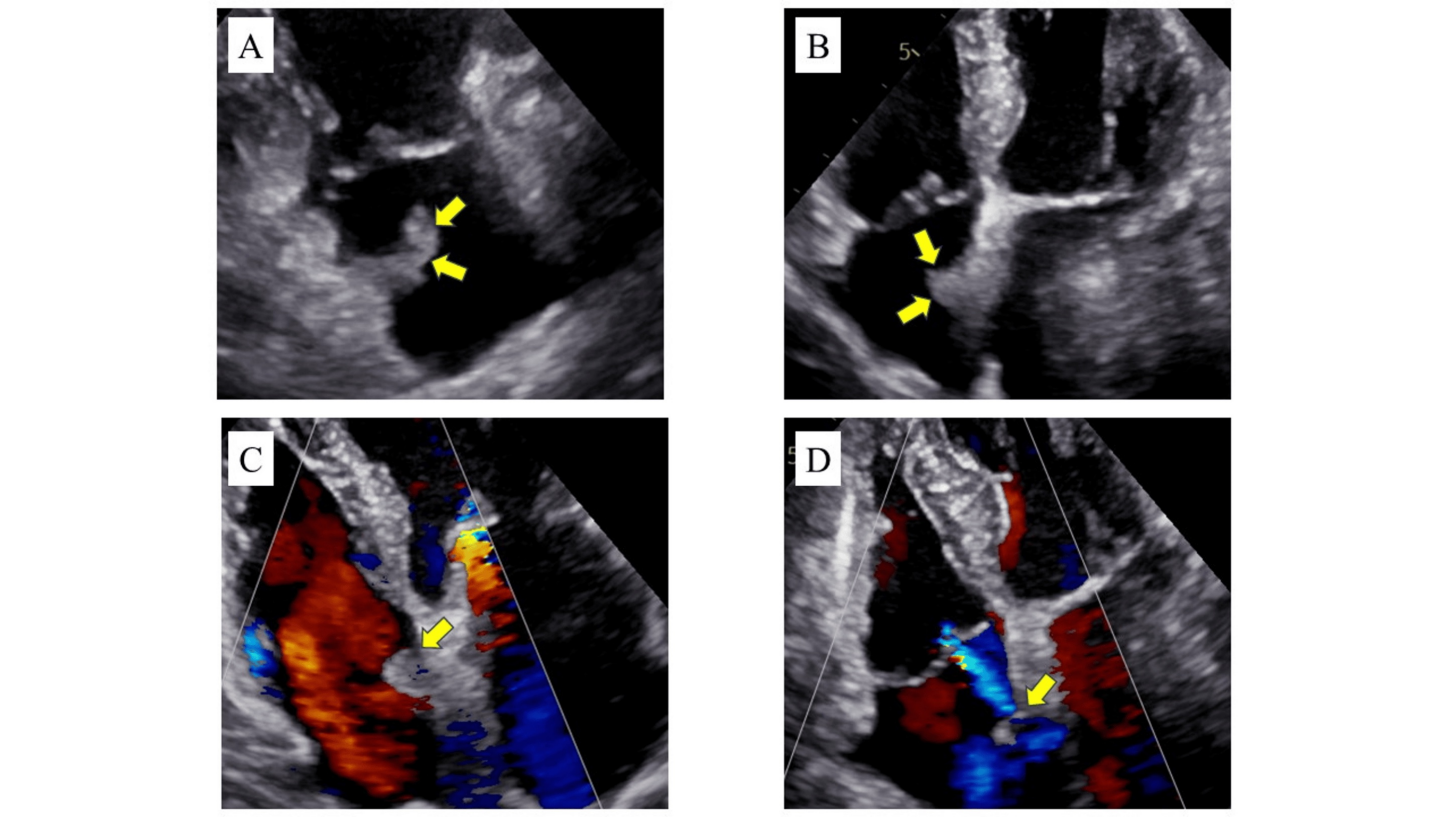
Preoperative TTE (A-D) TTE revealed that the tumor (yellow arrows), measuring 13 × 9 mm, was attached to the right atrial septum, highly mobile, well-defined with smooth edges, and exhibited homogeneous internal echogenicity without blood flow within the tumor. No interference with the tricuspid valve which could cause blood flow acceleration or severe regurgitation was detected. No other significant valvular diseases were noted. TTE: transthoracic echocardiography.

**Figure 2:**
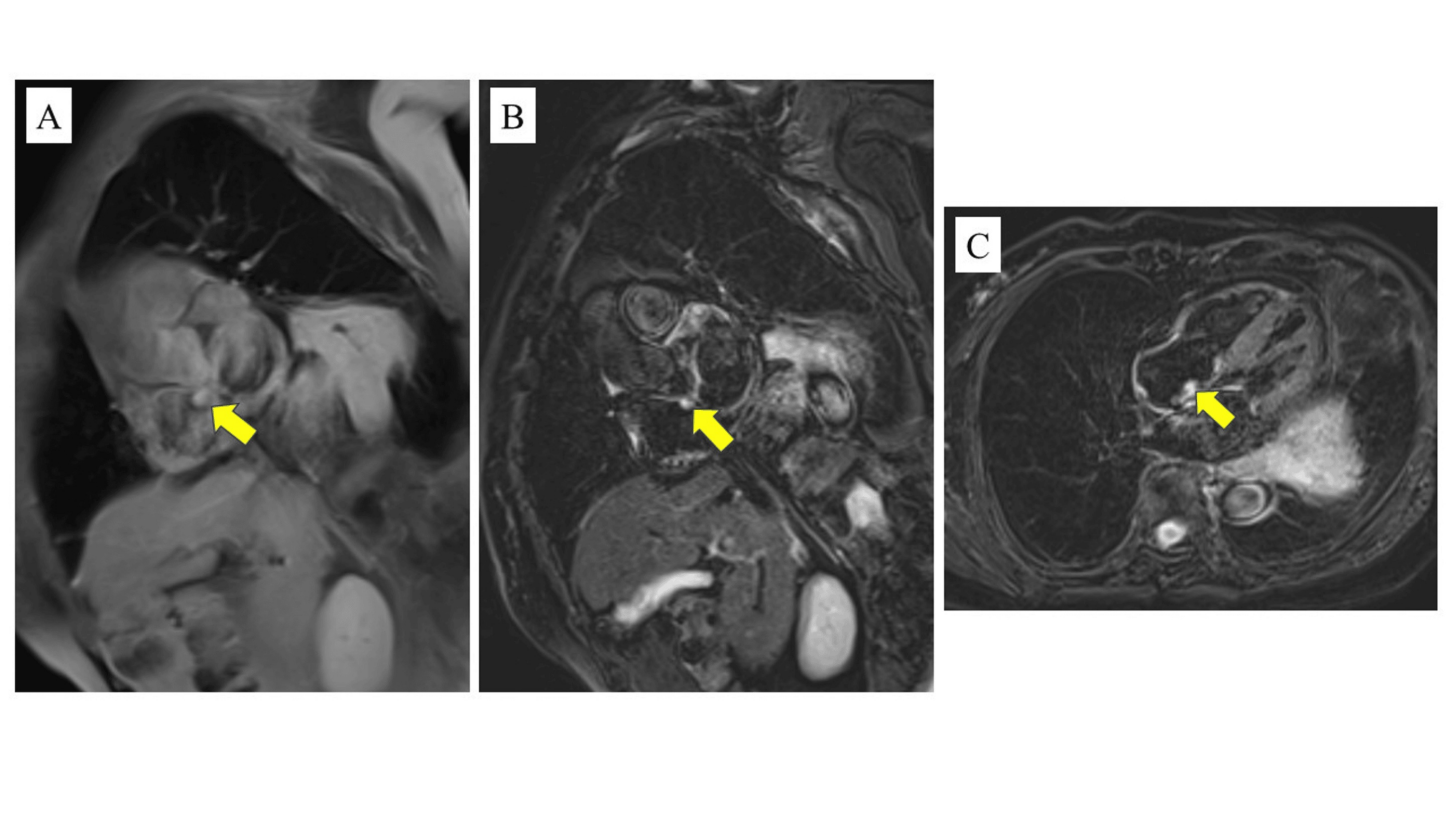
Preoperative CMR imaging CMR imaging revealed isointensity (yellow arrow) upon T1-weighted imaging (A), high signal intensity (yellow arrow) upon T2-STIR (B), and distinct enhancement (yellow arrow) after contrast administration (C). CMR: cardiac magnetic resonance, STIR: short tau inversion recovery.

A tumor was incidentally detected in the right atrium of an 83-year-old woman with advanced right lung cancer during preoperative TTE. The tumor, measuring 13 × 9 mm, was attached to the right atrial septum, highly mobile, well-defined with smooth edges, and exhibited homogeneous internal echogenicity without blood flow. No significant valvular diseases were observed (Figure 1). CMR imaging revealed isointensity on T1-weighted imaging, high signal intensity on T2-weighted short tau inversion recovery (STIR) imaging, and distinct enhancement after contrast administration (Figure 2). Computed tomography (CT) showed no distant metastasis, and positron emission tomography (PET)-CT demonstrated no abnormal accumulation outside the right lung cancer, ruling out malignancy of the cardiac tumor. Based on these multimodality imaging findings, a myxoma originating from the right atrial septum was suspected.

The five-year survival rate for lung cancer was expected to exceed 80%. Considering the tumor’s high mobility, early surgical resection, similar to the approach for lung cancer, was considered essential to prevent embolism. However, simultaneous cardiac and lung surgery was deemed high risk due to invasiveness (prolonged operation time and increased blood loss), given that the patient was of advanced age and had limited activities of daily living (ADLs). As the tumor was small and suspected to be benign, we decided to prioritize right upper lobectomy for lung cancer. Tumor resection via MICS was planned for after the patient recovered from lung surgery and regained surgical tolerance, to minimize perioperative risks.

**Figure 3:**
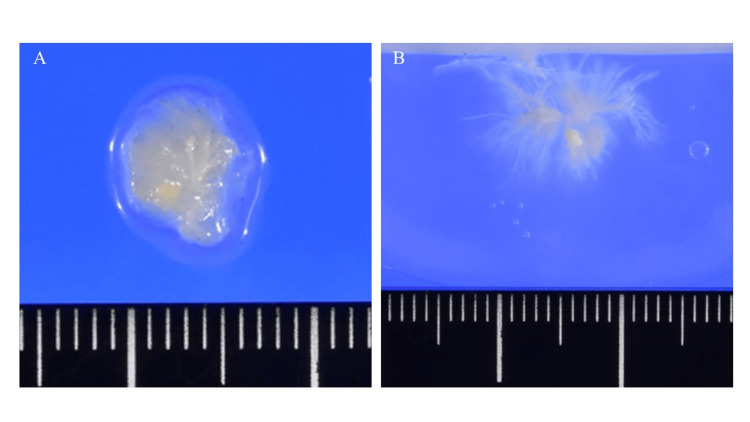
Resected tumor (A) Resected tumor. (B) When the tumor was immersed in water, numerous yellow-white projections extended from it, resembling a sea anemone, a characteristic feature of PFEs. PFE: papillary fibroelastoma.

Under general anesthesia with differential lung ventilation, a 7-cm skin incision was made at the fourth intercostal space, below the right breast. Mild adhesions from the right upper lobectomy were observed. Cardiopulmonary bypass was performed via the right femoral artery, right internal jugular vein, and femoral vein. After cardiac arrest was established, the right atrium was opened, revealing a colorless tumor attached to the upper left region of the fossa ovalis. The tumor was excised along with a portion of the right atrial septum. When immersed in water, the tumor exhibited numerous yellow-white projections resembling a sea anemone, a characteristic feature of PFE (Figure 3).

The postoperative course was uneventful, and TTE revealed no residual tumor or atrial septal shunt. The patient was discharged nine days post-surgery, and no recurrence was detected at the six-month follow-up. Pathological examination of the excised tumor revealed papillary structures with a collagenous core covered by a single layer of flattened cells and an Alcian blue-positive mucinous matrix, consistent with a PFE.

## Discussion

Primary cardiac tumors are rare, with an incidence rate of 1.38 per 100,000 individuals per year in population studies [5]. PFEs, which are benign and primary cardiac tumors, are the second most common primary cardiac tumors following myxomas [2]. However, recent studies suggest that PFEs may be more prevalent than myxomas. Tamin et al. reported a frequency of 1 PFE per 1,100 echocardiograms, surpassing myxomas in prevalence [4]. They are small, highly papillary, pedunculated, and avascular tumors, coated with a single endothelial layer and containing fine elastic fibrils arranged in whorls within a hyalinized stroma [2]. Macroscopically, their characteristic sea anemone-like appearance can be easily confirmed by immersing the excised tumor in water [4].

The histogenesis of PFEs remains unclear. Proposed etiologies include neoplasms, hamartomas, organized thrombi, and unusual endocardial responses to infections or iatrogenic factors (such as radiation, surgery, and hemodynamic trauma) [6]. The most widely accepted theory is the microthrombus hypothesis, which suggests that small thrombi coalesce at valve margins, where minor endothelial damage occurs [7]. This theory is supported by the presence of fibrin, hyaluronic acid, and laminated elastic fibers, which are characteristic of routine thrombus formation.

PFEs slightly more commonly affect men (55%) than women and are most frequently found in patients in their 70s, in contrast to myxomas, which are more common in women in their 40s and 60s. In one large study, 84% of PFEs were located on the valvular surface, with the aortic valve being the most commonly affected (44%), followed by the mitral (35%), tricuspid (13%), and pulmonary (8%) valves. Outside the valves, PFEs most often originated from the left ventricle (9%), whereas only approximately 2% originated from the right atrium [2].

The advancement of cardiac multimodality imaging has improved the diagnostic accuracy for cardiac masses [3], which may explain why PFEs have become the most commonly diagnosed primary cardiac tumors [8]. TTE is the primary diagnostic imaging technique for evaluating cardiac masses, with a diagnostic accuracy exceeding 80%. A typical PFE appears as a well-demarcated, small, mobile, pedunculated, valvular, or endocardial mass that protrudes into the cardiac chambers during the cardiac cycle [9]. However, the accuracy of echocardiography may be accompanied by a poor acoustic window, the presence of artifacts, and operator variability and expertise [10].

CMR imaging plays a crucial role in confirming the presence of a cardiac mass owing to its larger field of view, superior contrast resolution, and unique ability to differentiate lesions based on tissue characteristics. Recent developments in magnet strength, surface-coil channels, rapid k-space sampling, post-processing methods, and tissue characterization techniques made CMR an invaluable tool for assessing complicated cardiac conditions. PFEs appear isointense on T1-weighted images and hyperintense on T2-weighted images. After the administration of gadolinium, PFEs typically exhibit little to no enhancement in early images but display increased enhancement in delayed images. This pattern of delayed enhancement is attributed to the collagen content of PFEs, which resembles that of infarcted scar tissue [11].

In our case, CMR imaging yielded isointensity on T1-weighted imaging, high signal intensity on T2-weighted STIR imaging, and distinct enhancement after contrast administration. These features are also observed in myxomas. Given the tumor’s location and the findings from TTE and CMR, a myxoma was the most likely diagnosis. However, had dynamic imaging been performed, a PFE might have been suspected based on contrast changes over time.

The primary symptom of PFEs is embolism, which can occur due to tumor collapse or the release of a fibrin thrombus from the tumor surface, potentially leading to transient ischemic attacks or stroke (17%), acute myocardial infarction (4%), or sudden death (3%) [2]. These symptoms are more commonly seen on the left side of the heart. In contrast, a PFE on the right side of the heart is often asymptomatic until it grows large enough to cause thrombi that obstruct intracardiac blood flow or fragments of the tumor or thrombi that intermittently dislodge into the pulmonary circulation, causing a pulmonary embolism [12].

The mobility of the PFE is the only independent risk factor for embolism and death. Therefore, for symptomatic patients with mobile PFEs, radical surgical resection is strongly recommended, regardless of anatomical location unless significant contraindications exist [2]. Patients who undergo surgical resection experience lower rates of cerebrovascular accidents and mortality compared to those who do not undergo surgery. In addition, the recurrence rate is only 1.6%, which underscores the effectiveness of this intervention [4]. The management of asymptomatic patients with incidentally detected small and immobile PFEs, particularly on the right side of the heart, remains a therapeutic dilemma. Since PFEs grow very slowly [13], surgery may be deferred for asymptomatic patients under close echocardiographic monitoring. Anticoagulation therapy can help prevent thrombus formation [14].

In our case, although the patient was asymptomatic and the tumor was small, its high mobility warranted surgical resection. In addition, resection via MICS was deemed a suitable approach owing to the patient’s limited ADLs and the tumor location. Even though median sternotomy remains the primary surgical approach, recent studies suggest MICS provides favorable exposure and safe resection [15]. Tailoring the surgical approach to individual cases may be beneficial. Clear, standardized protocols for managing PFEs are lacking, and guidelines based on the accumulation of case reports and previous studies are eagerly awaited.

## Conclusions

We encountered a rare case of a PFE arising from the right atrial septum, which was successfully resected via MICS. The advancement of multimodality cardiac imaging has improved the diagnostic accuracy for PFEs, likely contributing to their emergence as the most commonly diagnosed primary cardiac tumors. Although PFEs are benign and often asymptomatic, especially on the right side of the heart, they can lead to fatal complications such as embolism. Therefore, early surgical resection should be considered for highly mobile tumors. Given its advantages in providing minimal exposure and ensuring safe resection, MICS may be a preferred approach, depending on the patient’s clinical profile, tumor characteristics, and location.
